# Metal ions and nanometallic materials in antitumor immunity: Function, application, and perspective

**DOI:** 10.1186/s12951-023-01771-z

**Published:** 2023-01-19

**Authors:** Feiyang Shen, Yan Fang, Yijia Wu, Min Zhou, Jianfeng Shen, Xianqun Fan

**Affiliations:** 1 Department of Ophthalmology, Ninth People’s Hospital, Shanghai Jiao Tong University School of Medicine, Shanghai, 200025 China; 2 Shanghai Key Laboratory of Orbital Diseases and Ocular Oncology, Shanghai, 200025 China; 3 Institute of Translational Medicine, National Facility for Translational Medicine, Shanghai Jiao Tong University, Shanghai, 200240 China

**Keywords:** Metal ions, Nanometallic materials, Cancer immunotherapy, Nanotechnology, Tumor microenvironment

## Abstract

The slightest change in the extra/intracellular concentration of metal ions results in amplified effects by signaling cascades that regulate both cell fate within the tumor microenvironment and immune status, which influences the network of antitumor immunity through various pathways. Based on the fact that metal ions influence the fate of cancer cells and participate in both innate and adaptive immunity, they are widely applied in antitumor therapy as immune modulators. Moreover, nanomedicine possesses the advantage of precise delivery and responsive release, which can perfectly remedy the drawbacks of metal ions, such as low target selectivity and systematic toxicity, thus providing an ideal platform for metal ion application in cancer treatment. Emerging evidence has shown that immunotherapy applied with nanometallic materials may significantly enhance therapeutic efficacy. Here, we focus on the physiopathology of metal ions in tumorigenesis and discuss several breakthroughs regarding the use of nanometallic materials in antitumor immunotherapeutics. These findings demonstrate the prominence of metal ion-based nanomedicine in cancer therapy and prophylaxis, providing many new ideas for basic immunity research and clinical application. Consequently, we provide innovative insights into the comprehensive understanding of the application of metal ions combined with nanomedicine in cancer immunotherapy in the past few years.

**Graphical Abstract:**

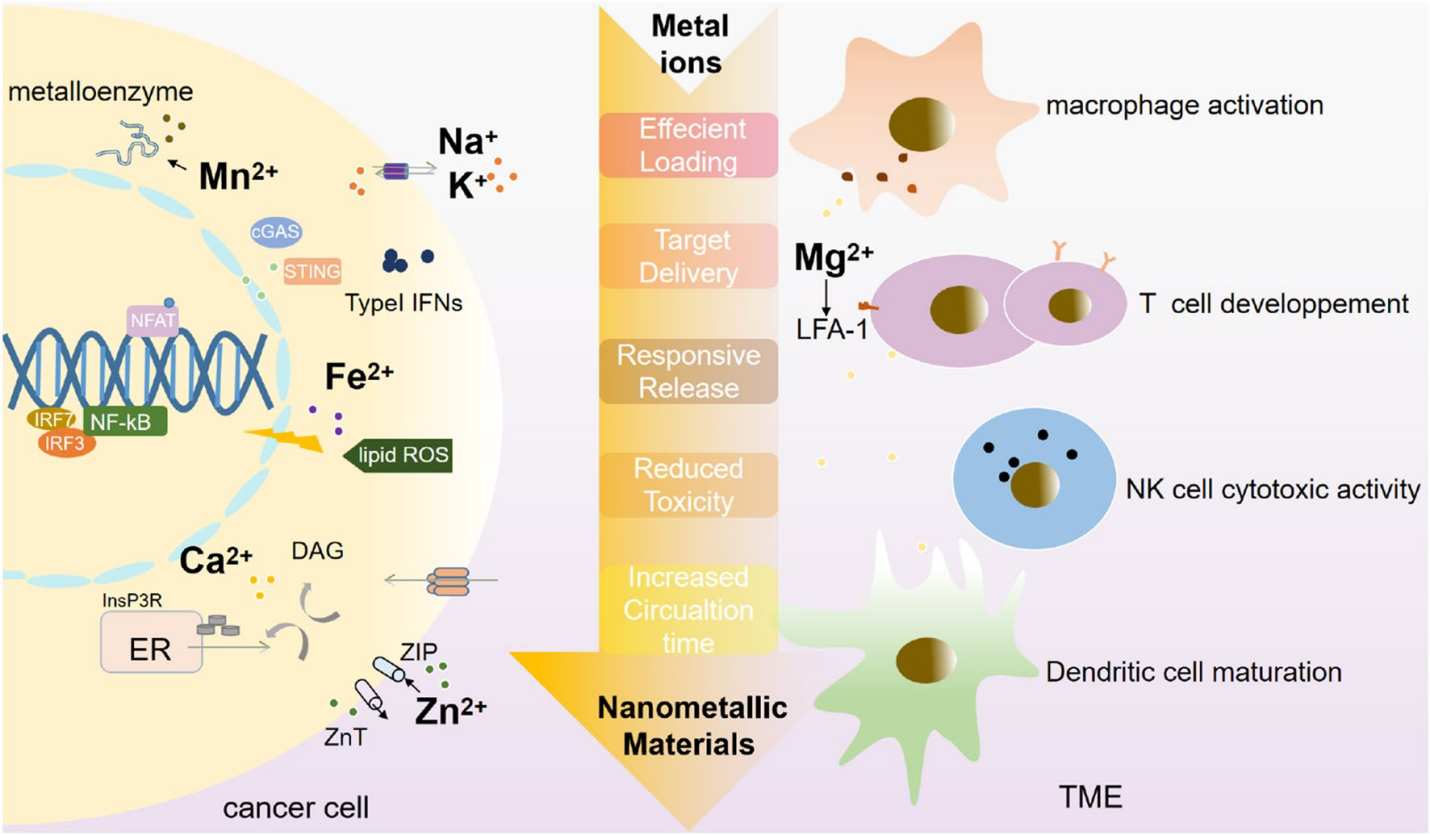

## Introduction

Because of its high mortality rate and limited treatment modalities, cancer has become a public health threat. As a result, some novel cancer therapies, including immunotherapy, photodynamic therapy (PDT), and photothermal therapy (PTT), are emerging in clinical practice and trials [1]. Cancer immunotherapy has attracted increasing attention due to its ability to induce powerful immune activation and persistent immune memory [2]. As many immune modulators or activators have been investigated, metal ions have demonstrated their superiority with high efficiency and ideal biocompatibility [3].

Metal ions can serve as elements that improve anticancer immunity and exert the function of cancer clearance. With the deepening understanding of the function of metal ions in cancer immunotherapy, a new term, “metalloimmunology”, was proposed in 2020 by Jiang et al. [4], and “cancer metalloimmunotherapy” was subsequently described by James J. et al. in 2021 [5]. The fewer side effects, feasible accumulation process and relative sensitivity to cancer cells indicate the advantages of metal ions for cancer immunotherapy.

In recent years, the development of nanotechnology has provided novel opportunities for the clinical application of tumor immunotherapy [6]. Multifunctional nanocarriers for tumor immunotherapy show various advantages, such as targeted delivery of immune activators to immune cells, thermal sensitivity systems of PTT/PDT, and reduction of side effects [7].

Nanometallic materials have become a promising delivery system because of their nanocrystalline strengthening effect, high photoabsorptivity, high surface energy, and single magnetic region performance [8]. Simultaneously, nanoparticulate delivery systems (NDs) are widely applied in tumor treatment because of their excellent efficiency [9].

Since the systemic application of metal ions may have toxic side effects [10], functionalized nanoparticles are needed to carry various metal ions directly into target cells. Some metal nanoprobes inherently reflect local and systemic information, which allows the integration of nanodelivery and nanobioimaging technologies in cancer metalloimmunotherapy [6, 11]. This combination of “nanometalloimmunotherapy” shows significant potential for facilitating precise drug delivery and synergistic effects [11, 12]. Moreover, nanometalloimmunotherapy overcomes the inherent limitations of traditional immunotherapy [13] and the drawbacks of metal ion-based antitumor therapeutics, including the short circulation time, low target selectivity, and systematic toxicity [14], exhibiting extraordinary practical potential by eliminating barriers to immunology and other fields [15].

## The general function of metal ions in tumorigenesis and antitumor immunity

Metal ions are essential for cellular life because they participate in many fundamental biological processes, such as membrane excitability, signal transduction, metalloprotein catalysis, and cell death [16]. A slight change in their concentration or cellular compartments may elicit metabolic dysfunction and disrupt the homeostasis of metal ions [17, 18]. Since the late 1980s, the application of metal ions has become especially prominent in cancer treatment because they participate in many cancer hallmarks, such as unrestricted proliferation, evasion of apoptosis, tissue invasion, and metastasis [19–22].

In addition to directly influencing cancer cell metabolism, metal ions contribute to cancer therapy by regulating hypoxia in the tumor microenvironment (TME) [7, 23–25]. The TME is characterized by a lower pH and higher levels of glutathione (GSH) and hydrogen peroxide (H_2_O_2_), which leads to the accumulation of immunosuppressive cells, including regulatory T cells (Tregs) and myeloid-derived suppressor cells (MDSCs) [26, 27]. Metal ions can reverse the low response of conventional cancer immunotherapy by inducing redox reactions serving as reducing agents with simultaneous oxygen production [14, 28, 29].

**Fig. 1:**
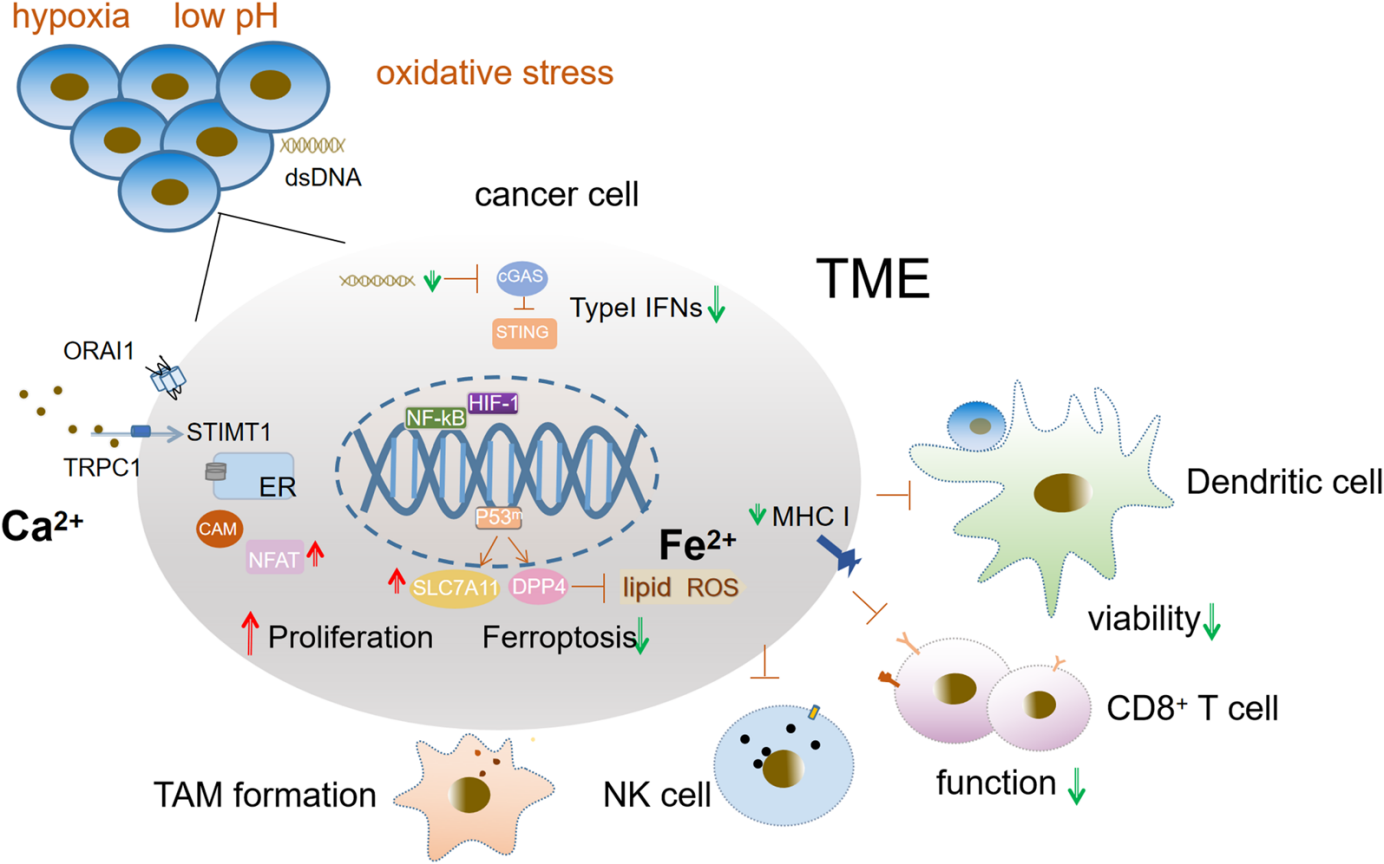
Metabolism of metal ions in cancer cells related to tumorigenesis

At the same time, metal ions are essential for the activation and proliferation of immune cells in the TME [30]. On the one hand, metal ions can promote innate immunity by enhancing the presentation capacity of dendritic cells (DCs) and macrophages and the cytotoxicity of natural killer (NK) cells [31–33]. On the other hand, metal ions can stimulate the activation and proliferation of adaptive immune cells, including CD8^+^ T cells (Fig. 1) [34], which exert a dominant function in antitumor immunity [35].

In the TME, the metabolism of metal ions in cancer cells and immune cells plays a major role in the development and metastasis of cancer. For instance, colon cancer cells display enhanced store-operated Ca^2+^ entry (SOCE), whose molecular players include ORAI1 and TRPC1 channels and stromal interacting molecules (STIMs) 1 and 2. In addition to abnormal growth, cancer cells resist cell death, such as ferroptosis, in a process involving metal ions. The disturbed signaling network of metal ions determines the features of cancer cells and their surroundings and supports the formation of tumor-associated macrophages (TAMs) and the dysfunction of lymphocytes.

## Metal ions participate in innate and adaptive immune activation in cancer

### Metal ions with innate immune activation ability in the TME

Innate immunity involves a wide range of immune cells, such as DCs, macrophages, and NK cells [36]. Although innate immune cells can detect tumors and induce and amplify adaptive immune responses, the immunosuppressive microenvironment at the tumor bed represses their functions [37]. Metal ions have been revealed to promote the pathogen‒host interaction and activate the cyclic GMP-AMP synthase-stimulator of interferon genes (cGAS-STING) pathway and NLRP3 (NOD-, LRR- and pyrin domain-containing protein 3) inflammasomes, which can serve as innate immunity activators and boost anticancer immunity.

#### Fe^2+^/Zn^2+^/Cu^2+^ modulates the pathogen‒host interaction

Dysbiosis is considered a contributing factor in the origin and development of colon cancer because disturbed host-microbe interactions may lead to chronic inflammation. Metal ions have been confirmed to be essential for intestinal inflammation recovery [38, 39].

Mouse experiments have revealed that intestinal stimulation by microbes can induce type 2 conventional DCs (cDC2s) to release hepcidin, which is a key regulator of systemic Fe^2+^ homeostasis. At the same time, cDC2s play a dominant role in human intestinal inflammation and local mucosal repair by promoting ferroportin-mediated Fe^2+^ sequestration from intestinal macrophages that have phagocytosed erythrocytes [40]. Colorectal cancers (CRCs) often result in intestinal bleeding and hence anemia, and limited Fe^2+^ levels in the intestinal lumen can reduce tissue gut infiltration and therefore promote intestinal repair [41, 42].

Zn^2+^ is an essential but toxic microelement in high concentrations and can serve as an antimicrobial strategy for Mycobacterium [43, 44], which can provoke chronic inflammation related to cancer pathogenesis [45]. Meanwhile, the P1B-type ATPase CtpG (Rv3270) was recently identified as a Zn^2+^ efflux transporter via the CmtR-CtpG-Zn^2+^ regulatory pathway that enhances mycobacterial resistance to Zn^2+^ toxicity because the accumulation of Zn^2+^ can contribute to ROS detoxification in mycobacterial cells [46].

The intestinal microbiota can also affect the therapeutic effects of antineoplastic agents, such as disulfiram, whose anticancer effect is enhanced by combining with antibiotics and Cu^2+^ by significantly reducing the expression of phosphorylated protein kinase B (p-AKT)/protein kinase B (AKT), Toll-like receptor 4 (TLR-4), and phosphorylated nuclear factor kappa-B (p-NFκB)/NFκB in tumors [39].

#### Zn^2+^/Mn^2+^/Co^2+^ activates the cGAS-STING signaling pathway

The stimulator of interferon genes (STING) pathway has been proven to play critical roles in the initiation of antitumor immunity. In particular, the STING pathway can ameliorate the immunosuppressive network in “cold” tumors [37, 38]. In brief, cyclic GMP-AMP synthase (cGAS) detects damage-associated double-stranded DNA (dsDNA) in the cytosol and catalyzes the generation of cyclic GAMP (cGAMP), which serves as the second messenger to activate STING and induce type-I interferons (IFNs) [47, 48]. As a result of chromosomal instability, cytosolic dsDNA in cancer cells elicits cancer immunogenicity via cGAS-STING pathway activation, which originates primarily from the vulnerable membrane of micronuclei [49]. To escape from the suppressive signaling axis, cancer cells inhibit the cGAS-STING pathway by reducing the expression of cGAS and STING and co-opting STING-dependent DNA sensing [50].

In addition to abnormal dsDNA, some metal ions, such as Mn^2^, Zn^2+^, and Co^2+^, have been recently shown to be activators of the cGAS-STING pathway [51, 52]. In 2018, J. Chen et al. proved that Zn^2+^, Mn^2+^ and Co^2+^ can significantly promote the combination activity of cGAS in vitro, which could induce DNA-induced cGAS phase separation even at low concentrations [53]. Moreover, Zn^2+^ can largely enhance cGAS activity by binding to cGAS and stabilizing the cGAS-DNA complex [47].

**Fig. 2:**
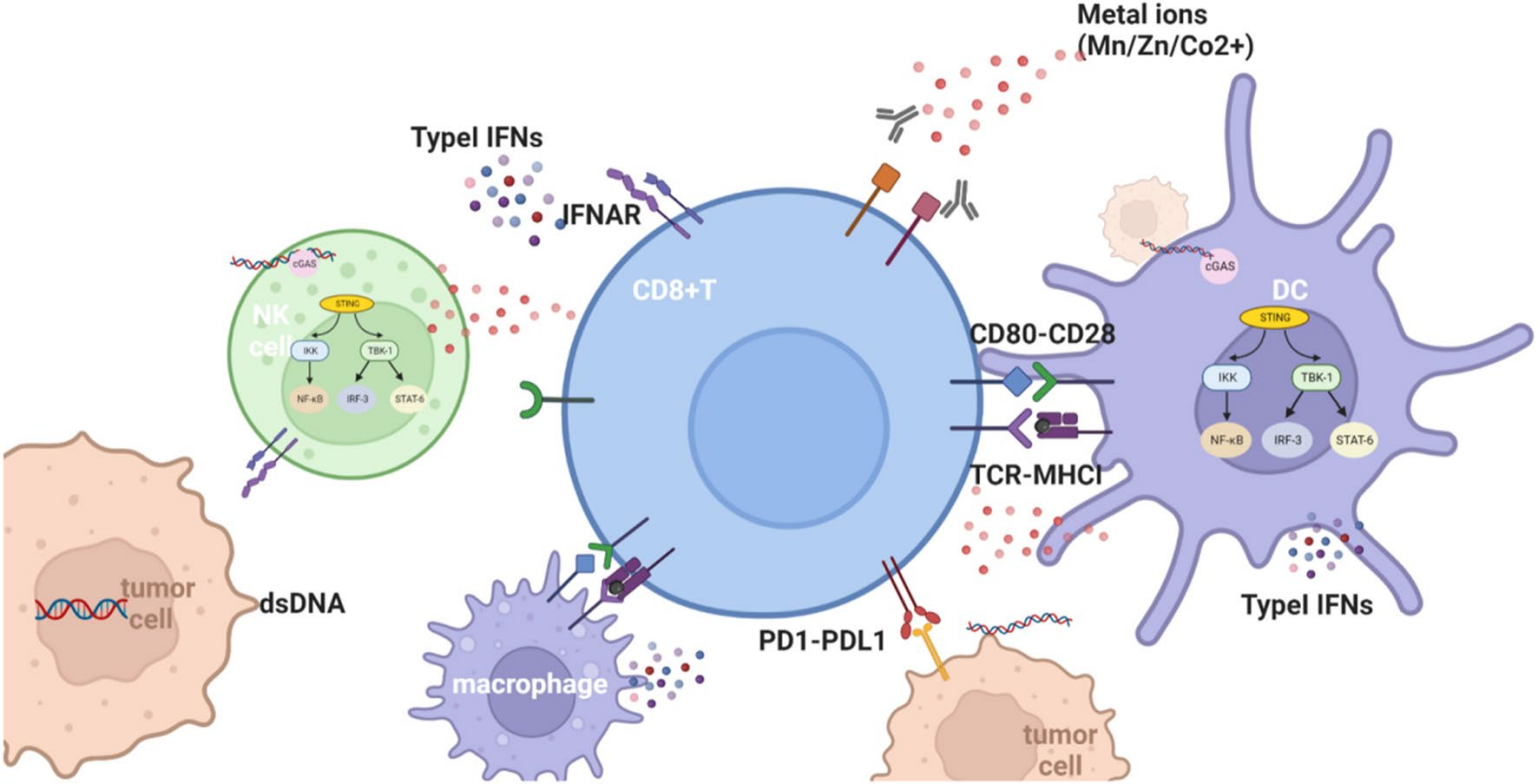
Mn^2+^/Zn^2+^/Co^2+^ is indispensable for host defense triggered by cytosolic dsDNA, which activates the cGAS-STING signaling axis and produces type-I IFNs

Wang et al. revealed that Mn^2+^ can replace Mg^2+^ as a cofactor and markedly enhance activation of the cGAS-STING signaling axis not only by sensitizing cGAS and its adaptor STING but also by largely increasing the STING-cGAMP binding affinity [53]. In addition, an increasing number of studies have shown that Mn^2+^ can potentiate the STING pathway by amplifying cGAS-STING recognition in immune cells via a direct interaction with tumor cells mediated by Mn^2+^ [53]. Additionally, Mn^2+^ promotes cross-presentation between DCs and CD8^+^ T cells and the cytotoxic function of NK cells and cytotoxic T lymphocytes (CTLs). Among cancer patients with multidrug resistance or advanced metastatic solid tumors, Mn^2+^ rescues the clinical efficiency of some cancer treatments such as PD-1/PD-L1 therapy and greatly improves their prognosis [51, 54] (Fig. 2).

Mn^2+^, Zn^2+^ and Co^2+^ can contribute to cGAS binding to STING at the endoplasmic reticulum (ER), and this complex translocates from the ER to Golgi compartments. STING serves as a signaling platform for TANK-binding kinase 1 (TBK1) and IkappaB kinase (IKK). TBK1 phosphorylates STING, which in turn recruits interferon regulatory factor 3 (IRF3) for TBK1-mediated phosphorylation, which stimulates the transcriptional expression of IFNs and other immune-stimulatory genes.

#### Ca^2+^/K^+^/Na^+^ activates the NLRP3 inflammasome

Chronic inflammation and persistent infection contribute to different malignancies, such as melanoma, since some inflammasomes, including NLRP3 inflammasomes, have a pathogenic role in triggering a broad range of cellular perturbations, such as damage-associated molecular patterns (DAMPs) [55]. NLRP3 inflammasomes provoke the release of the pro-inflammatory cytokines IL-1β and IL-18 in the foundation of caspase-1, which is considered as a promoting factor for cancer development, invasion, metastasis and chemoradioresistance [56, 57]. Metal ions such as Ca^2+^/K^+^/Na^+^ can activate the NLRP3 inflammasome and caspase-1, thereby inducing high levels of bioactive IL-1β and tumorigenesis [58, 59].

The increase in [Ca^2^^+^]e detected by monocytes can activate the phosphatidyl–inositol/Ca^2+^ pathway, which in turn leads to NLRP3 activation [60, 61]. At the same time, as Ca^2+^ uptake through the mitochondrial Ca^2+^ uniporter (MCU) can enhance the phagocytosis-dependent NLRP3 inflammatory response, the amplified release of IL-1β aggravates tissue damage via inefficient inflammatory pathways induced by mitochondrial DAMPs, which promotes neoplastic disorders [62, 63].

**Fig. 3:**
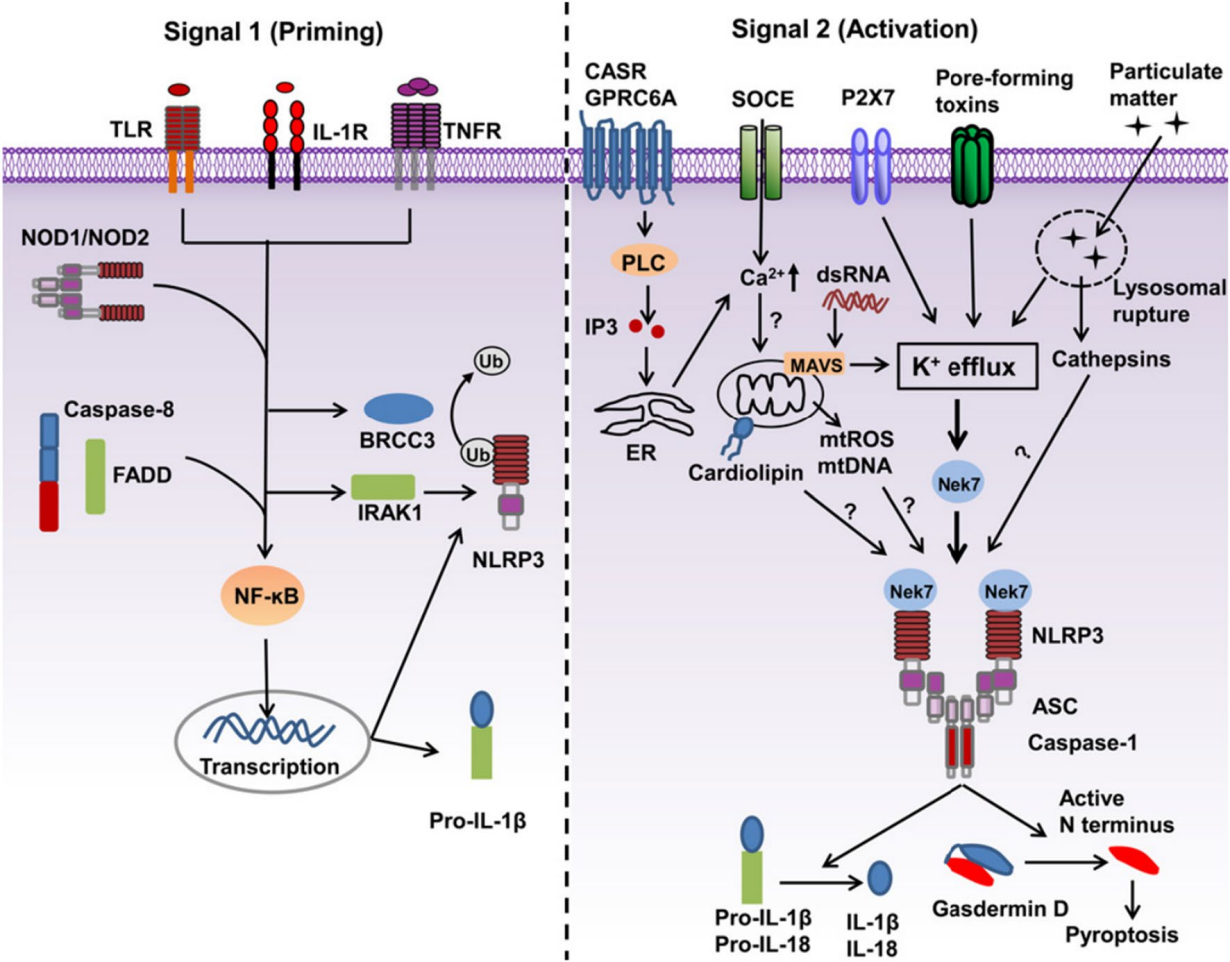
A two-signal model for NLRP3 inflammasome activation [55] @Copyright 2022, Elsevier Ltd

NLRP3 activation is also determined by membrane permeability to K^+^ and Na^+^. In particular, the reduction in [K^+^]i is necessary and sufficient for caspase-1 activation [57]. K^+^ efflux is widely accepted as a conjoint point in the activation of the NLRP3 inflammasome since internalization by phagocytosis can induce lysosomal membrane damage and trigger the opening of membrane pores permeable to K^+^ [59, 63]. In addition, Na^+^ influx can modulate NLRP3 activation independent of K^+^ efflux but is not an absolute requirement. Recently, the correlation of enhanced epithelial sodium channel (ENa_C_)-dependent Na^+^ influx with exacerbated NLRP3 inflammasome activation was observed in a monogenic disease accompanied by increased K^+^ efflux [58] (Fig. 3).

### Metal ions with adaptive immune activation ability in the TME

Adaptive immune responses are crucial for the recognition and elimination of infective and neoplastic cells [37]. Homeostasis of metal ion metabolism is essential for the differentiation and function of immune cells, especially for the main adaptive immune effector CD8^+^ T cell [64]. Considering that CD8^+^ T-cell exhaustion within the TME usually causes a low response to cancer immunotherapy [26], the regulation of metal ions contributes to the infiltration of CD8^+^ T cells, thus reversing local immunosuppression [65].

#### K^+^ is crucial for CD8^+^ T-cell function and stemness

Studies have shown that the increase in extracellular K^+^ impairs Akt-mTOR phosphorylation driven via TCR and facilitates subsequent effector processes, which are dependent on the activity of the serine/threonine phosphatase PP2A [65, 66]. Overexpression of the K^+^ channel Kv1.3 in melanoma-bearing mice can increase K^+^ efflux in tumor-specific T cells and enhance effector functions, thus promoting tumor clearance. The elimination of K^+^ from T cells restores their ability to attack cancer [67]; as a result, K^+^ can act as an ionic checkpoint blocking T-cell function in cancer immunotherapy.

Recently, an increasing concentration of K^+^ in the TME has been proven to promote the persistence and stemness of tumor-infiltrating lymphocytes (TILs) through functional caloric restriction, which promotes autophagy and metabolic reprogramming [68]. Increased [K^+^]e can selectively induce mitochondrial acetyl-CoA (AcCoA) synthetase 1, which drives metabolically abundant oxygen utilization for antitumor T cells [69]. As a result, K^+^ efflux improves T-cell persistence in tumor-bearing mice, which enhances tumor clearance and mouse survival time [70]. The increase in [K^+^] in the TME can reversibly and durably cultivate the expansion of T cells in vitro, indicating its application potential as an adoptive immunotherapy modulator.

#### Mg^2+^ regulates CD8^+^ T-cell effector function via LFA-1

Leukocyte function-associated antigen 1 (LFA-1) is an integrin that participates in T cell activation via immune synapse formation as well as in leukocyte trafficking and extravasation [71]. LFA-1 conformational changes are mediated by the T cell antigen receptor (TCR) signal stimulation, which is regulated by the binding of Mg^2+^ to metal-ion-dependent adhesion sites (MIDAS) [72]. This demonstrates that Mg^2+^ can affect T cell function by modulating proximal and distal signaling activity, such as focal adhesion kinase (FAK) and extracellular signal-regulated protein kinase 1/2 (ERK1/2) phosphorylation, respectively [73].

Mechanically, the extension and conformational opening of LFA-1 is regulated by Mg^2+^ on activated T cells, which is essential for FAK phosphorylation, calcium flux, and downstream effector functions in T-cell blasts [74]. As Mg^2+^ is an essential cofactor for DNA damage repair enzymes, deficiency of Mg^2+^ results in the accumulation of DNA damage and contributes to cancer progression. A low concentration of Mg^2+^ is related to TCR signal suppression, which inhibits T-cell proliferation and contributes to T-cell exhaustion [65].

#### Ca^2+^ promotes CD8^+^ T-cell activation by CD3 phosphorylation

Ca^2+^ can bind directly to anionic phospholipids, serving as a modulator for membrane protein function. The ionic interactions between positively charged CD3 cytoplasmic domains and negatively charged phospholipids in the plasma membrane regulate the activation of TCR. Since increasing the [Ca^2+^]i concentration can induce the dissociation of CD3 from the membrane and the solvent exposure of tyrosine residues, CD3 tyrosine phosphorylation can be significantly enhanced by Ca^2+^ influx. Instead of initiating CD3 phosphorylation, this Ca^2+^ regulatory pathway can amplify and sustain CD3 phosphorylation, which enhances T-cell sensitivity to some antigens, such as major histocompatibility complex (MHC) [75].

Ca^2+^ influx can increase the accessibility of the immunoreceptor tyrosine-based activation motifs (ITAMs) in the CD3 cytoplasmic domains to lymphocyte-specific protein tyrosine kinase (Lck) phosphorylation and can thus trigger the signaling cascade to activate T cells. The robust Ca^2+^ influx can compete with phospholipids to help CD3 ITAMs release from the inner leaflet of the plasma membrane. TCR signal transduction is crucial to T-cell activation in immune responses; therefore, the participation of Ca^2+^ in T-cell-engaging therapies deserves to be further explored [76, 77].

## The application of metal ion-based antitumor therapeutics and the perspective of nanometallic materials in cancer immunotherapy

Metal ions exert pivotal functions in cancer immunology by modulating cellular metabolism and the TME, which enhance the efficacy of cancer immunotherapy and demonstrate their important clinical potential through delicate mechanisms (Table 1).

**Table 1:** The important roles of metal ions in antitumor immunology

Metal ion	Location	↑↓	Functions	Mechanisms	Applications	Refs.
K^+^	TME	↑	T-cell effector dysfunction; CD8^+ ^T-cell stemness preservation	Functional caloric restriction; autophagyautophage↑; effector programs↓	Adoptive cell transfer therapy	[68]
Mn^2+^	TME	↑	NK cell activation; DC function↑; CD8^+^ T proliferation↑ differentiation↑; M1 polarization	Sensitizes cGAS and its adaptor STING; STING-cGAMP binding affinity; type-I IFN ↑	Innate immunity activator	[5, 51]
Mg^2+^	TME	↑	T-cell activation; T-cell effector function↑; T-cell cytotoxicity↑; Mg^2 +^ influx acts as second messenger in TCR signaling	Increases LFA-1 outside-in signaling; directly interacts with IL-2-inducible T-cell kinase (ITK) promoting its activation	Combined with PD-1 blockade; Target Mg^2+^ transporters	[73, 96]
Fe^2+/3+^	Cancer cells	↑	Immunotherapy-activated CD8^+^ T cells enhance ferroptosis-specific lipid peroxidation in cancer cells	CD8^+^ T cells secrete IFNγ; downregulates the expression of SLC3A2 and SLC7A11	T-cell-promoted tumor ferroptosis + checkpoint blockade	[97]
Ca^2+^	CD8^+^ T cells	↓	Activation of CD8^+^ T cells and NLR3 inflammasomes; NK cells function↑	CD3 phosphorylation↑; TCR signal transduction; T-cell sensitivity↑	Store-operated Ca^2+^ entry (SOCE) block	[98, 99]
Zn^2+^	T lymphocytes	↑	Boosts immune functions; targets T-cell metabolism; immune surveillance	Increases in T -cell receptor-derived excision circles (TRECs) and CD4^+^ naïve lymphocytes	Zinc supplementation	[100]
Cu^2+^	Cancer cells	↓	Modulates PD-L1 expression; cancer immune evasion; CD8^+^ T, NK cells↑	Inhibits phosphorylation of STAT3 and epidermal growth factor receptor (EGFR); promotes ubiquitin-mediated degradation of PD-L1	Copper chelators	[101]
Pt^2+^	TME	↑	Induces increases in antigen processing machinery (APM) component expression; upregulation of immune checkpoints or impairment of T-cell function	Enhances antigen presentation and T-cell killing; increases tumor cell expression of PD-L1; impair T-cell function	Cisplatin; combination with immune checkpoint antibody	[102, 103]
Au^+/3+^	Immune cells	↑↓	Stimulates the activation and proliferation of T as well as B cells; promotes the T-cell-based anticancer immunity cycle via DC	Activates TLR3 signaling; stimulates immune cells to secrete key inflammatory cytokines; inhibits the DNA binding activity of NF-κB	Aurothioglucose; Auranofin; sodium aurothiomalate; HAuCl_4_ [Au(III)]; gold sodium thiomalate	[94]

↑: upregulation; ↓: downregulation

### Traditional therapy: Metallodrugs participate in antitumor immunity

Cis-diamminedichloroplatinum(II), known as cisplatin (Pt(NH_3_)_2_Cl_2_), is a first-line medicine for a broad range of cancers, such as lung, head, and neck cancer [78], and, which induces tumor-specific cytotoxicity based on structural lesions [79]. In addition to the induction of tumor cell apoptosis, cisplatin has been proven to optimize immune checkpoint blockade by increasing PD-L1 expression in non-small cell lung cancer [80], which indicates that cisplatin-based neoadjuvant chemotherapy could improve the clinical effectiveness of PD-1/PD-L1 treatment.

Sustained exposure to MnCl_2_ enhances humoral immunity. Nearly 30 years ago, intraperitoneal injection of MnCl_2_ was shown to enhance the activity of murine NK cells, which is probably mediated by the production of type I IFNs [81]. In addition, intratumor injection of MgCl_2_ was recently proven to enhance cellular immunity by regulating CD8^+^ T-cell activation and cytotoxicity [73]. In addition, magnesium supplementation has the potential to combat cisplatin resistance [82] and to modulate inflammatory factors, such as tumor necrosis factor (TNF-α) [83].

It was considered that sustained exposure to MnCl_2_ enhances humoral immunity. Nearly 30 years ago, intraperitoneal injection of MnCl_2_ was shown to enhance the activity of murine NK cells, which is probably mediated by the production of type I IFNs. In the meanwhile, intratumor injection of MgCl_2_ is recently proved to enhance the cellular immunity by shaping CD8^+^ T cells activation and cytotoxicity, meanwhile, magnesium supplementation has the potential of cisplatin resistance repair as well as inflammation factors modulation, like TNF-α.

### Targeted therapy: Metal ion channels and transporters regulate the TME

“Oncochannelopathy” is a term that was first proposed in 2018 by Prevarskaya et al. and refers to cancer hallmarks viewed as pathological states that are mainly caused by abnormal expression and/or dysfunction of certain ion channels [19]. Metal ion homeostasis and metabolism are highly regulated by distinct ion channels. Owing to the significant functions of metal ion channels in tumorigenesis and metastasis, many novel anticancer therapies have emerged that target metal ion transporters as agonists or inhibitors [84].

For Zn^2+^, the SLC39A (zrt/irt-like proteins; ZIP) family and SLC30A (cation diffusion; ZnT) Zn^2+^ family are widely investigated in cancer immunotherapy. Zinc transporters play an important role in B-cell development at different stages; for instance, SLC39A10 deficiency leads to a reduced population of both pro- and pre-B cells, and, SLC39A7 deficiency is a negative regulator of phosphatases with impaired BCR-dependent signaling in pre- and immature B cells [85, 86]. In addition, SLC39A6 in DCs and T cells can indirectly activate the TCR activation pathway, which leads to cell proliferation and cytokine production [87].Ladiratuzumab vedotin is a new drug of antibody drug conjugates (ADCs) targets for of SLC39A6, which is under evaluation in an ongoing open-label phase Ib/II trial.

SLC41A1 and SLC41A2 are two key transporters expressed in lymphoid cell lines and various immune cells, respectively [87]. SLC41A functions as a Mg^2+^/Na^+^ exchanger whose overexpression could partially repair the deletion of other Mg^2+^-permeable ion channels, such as TRPM7 (transient receptor potential cation channel subfamily M member 7), thus rescuing the reduction in cell growth and maintaining the normal growth of lymphocytes [88].

### Pyroptosis/ferroptosis/cuproptosis: Metal ions induce nonapoptotic cell death

Pyroptosis is a special type of inflammatory cell death that evokes a proinflammatory signal and stimulates tumor immunogenicity via gasdermin D (GSDMD). The first metal complex-based pyroptosis inducer was recently reported to be carbonic anhydrase IX (CAIX)-anchored ruthenium(I) photosensitizer CA-Re, which promoted the maturation and antigen-presenting ability of DCs and activated the T-cell-dependent adaptive immune response [89].

Ferroptosis is a type of Fe^2+^-dependent cell death that damages polyunsaturated fatty acid-containing phospholipids in cellular membranes mediated by lipid peroxidation (LPO) [90]. In recent years, increasing numbers of natural and synthetic drugs related to ferroptosis have been identified [91]. Conventionally, inhibition of GSH and glutathione peroxidase 4 (GPX4) expression leads to the accumulation of lipid peroxides, inducing ferroptosis. Innovatively, vaccination with early ferroptotic cancer cells is reported as an inducer of efficient antitumor immunity, which promotes the phenotypic maturation of bone marrow-derived dendritic cells (BMDCs) and elicits adaptive immunity activation [92].

A recent study revealed that Cu^2+^-dependent death is another type of cell death that relies on mitochondria by means of direct binding of copper to lipoylated components of the tricarboxylic acid (TCA) cycle. The subsequent lipoylated protein aggregation and iron-sulfur cluster protein loss leads to proteotoxic stress and cell death [93]. Many studies have noted that the cuproptosis-related gene (CRG) signature can predict immune characteristics in various cancers, which provides guidance for prognosis, clinicopathological features, immune characteristics, and treatment preference in precise and individual cancer strategies.

### Drawbacks for metal ion-based antitumor therapeutics and possible solutions

Metal ion-based complexes such as cisplatin are only effective against limited types of tumors and have a variety of serious side effects, such as gastrointestinal and nervous system toxicity and bone marrow suppression. Additionally, intrinsic and acquired drug resistance attenuate the effectiveness of these agents. [94] More importantly, systemic toxicity, short circulation time, and low target selectivity have hampered their clinical applications to a great extent. While the application of metal ions alone has many deficiencies, the clinical application of nanometallic materials showed predominant advantages, such as efficient loading, selective delivery and responsive release with longer circulation retention time. Cisplatin and copper ionophores represent an example.

A multifunctional nanogel (designated Valproate-D-Nanogel) was capable of reactivating cisplatin and enhancing early apoptosis. This nanogel can effectively inhibit cisplatin-resistant cancer through combined pathways and provides an effective approach for overcoming cisplatin resistance in cancer treatment [95]. With the development of nanotechnology, many copper ionophores, such as dithiocar-bamates (DTCs) and thiosemicarbazones (TSCs), have been developed and combined with cuproptosis inducers to increase the intracellular copper content and elicit efficient cancer cell damage, which can greatly improve the efficiency of cuproptosis induction and is regarded as a promising cancer strategy.

## Several breakthroughs of nanometallic materials in cancer immunotherapy

**Fig. 4:**
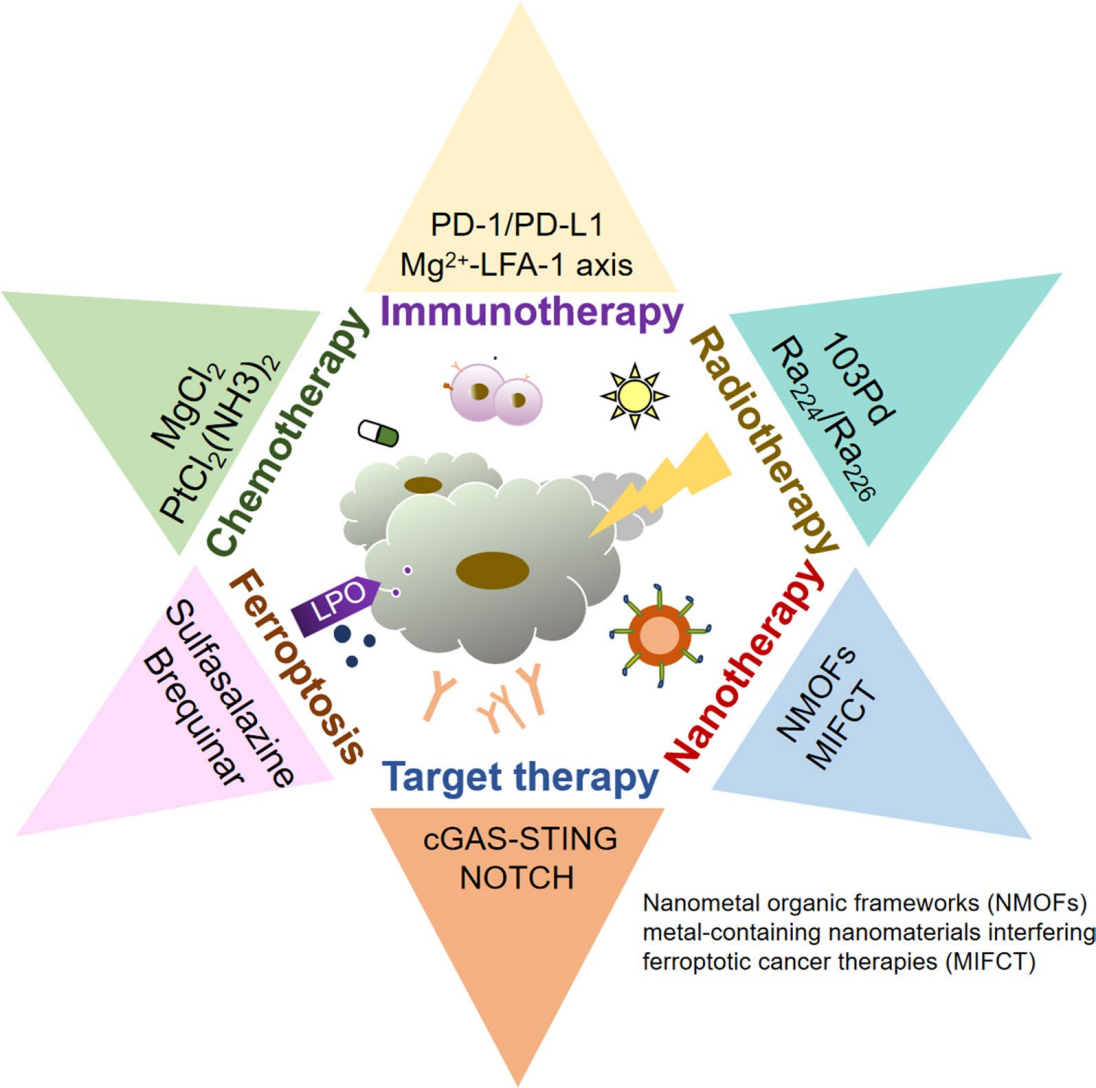
Various recent therapies for the treatment of cancer patients and the main techniques applied with metal ions of each treatment.

As we have mentioned above, many metal ions exert critical functions in antitumor immunity, and the synergistic strategy can enhance the effectiveness of both therapies and overcome their inherent limitations (Fig. 4) [11]. Considering that the combination of metal ions and nanotechnology possesses predominant advantages, including more stability, better efficiency at lower doses, and more importantly, fewer side effects than metal ions alone, we will introduce several essential metal ion nanoparticles applied in cancer immunotherapy in the last few years.

Cancer therapies can be combined with others, resulting in a better therapeutic effect and prognosis. Prospectively, the combination of different antitumor modalities will provide a new concept for cancer treatment and prevention.

### Manganese and MnO_2_-based nanoimmune activators

Manganese catalyzes the Fenton reaction but is also an activator of the cGAS-STING pathway in antitumor immunity. Amorphous porous manganese phosphate (APMP) NPs have been designed with a high responsive ability to TME, which could be loaded with doxorubicin (DOX) and phospholipid (PL)-coated hybrid nanoparticles to maintain stability in systemic circulation and activate the cGAS-STING signal pathway [104].

A metal-phenolic network (MPN)-based immune-active nanoparticle is newly synthesized by coordinating tannic acid with Mn^2+^ by electroporation and subsequent coating with CpG-oligodeoxynucleotides (CpG-ODNs) via hydrogen bonding. CpG-ODN-coated Mn-phenolic network nanoparticles can effectively internalize into macrophages and stimulate M1 polarization to promote the release of proinflammatory cytokines as an effective immune activator [28].

Manganese oxide (MnO_2_) nanomaterials are biodegradable with stable structures, excellent physiochemical features and biosecurity. MnO_2_ can catalyze H_2_O_2_ into dissolved O_2_ and consume GSH, which can react with ROS within the TME, serving as an enhancer for PDT-induced immunotherapy and photothermal agents (PTAs) [105]. In addition, Mn@CaCO_3_/ICG nanoparticles loaded with PD-L1-targeting small interfering RNA (siRNA), a type of TME-sensitive O_2_-generating nanosystem MnO_2_@Chitosan-CyI (MCC) and cGAS-STING activating MnIIIPC@DTX@PLGA@Mn^2+^@HA (MDPMH) nanoparticles have also been designed for precise individualized diagnosis and treatment of various tumors [106].

### Iron and iron oxide derivative-based nanoimmune activators

Iron oxide nanoparticles (IONPs) are ideal magnetic drug carriers with reasonable biodegradability and biocompatibility. IONPs have been applied as magnetic resonance imaging (MRI) contrast agents in cancer diagnosis, which could be guided to a specific area of interest [107, 108]. The magnetic hyperthermia triggered by IONPs can not only generate effective PTT/PDT but also enhance antigen presentation and DC maturation; at the same time, T lymphocytes can be recruited into lymph nodes, and immunosuppressive Tregs are depleted by the enhanced immune response. Thus, magnetic-responsive immunostimulatory nanoagents (MINPs) possessing magnet-guided and immunostimulatory properties have been designed and specifically eliminate primary and metastatic tumors [109].

**Fig. 5:**
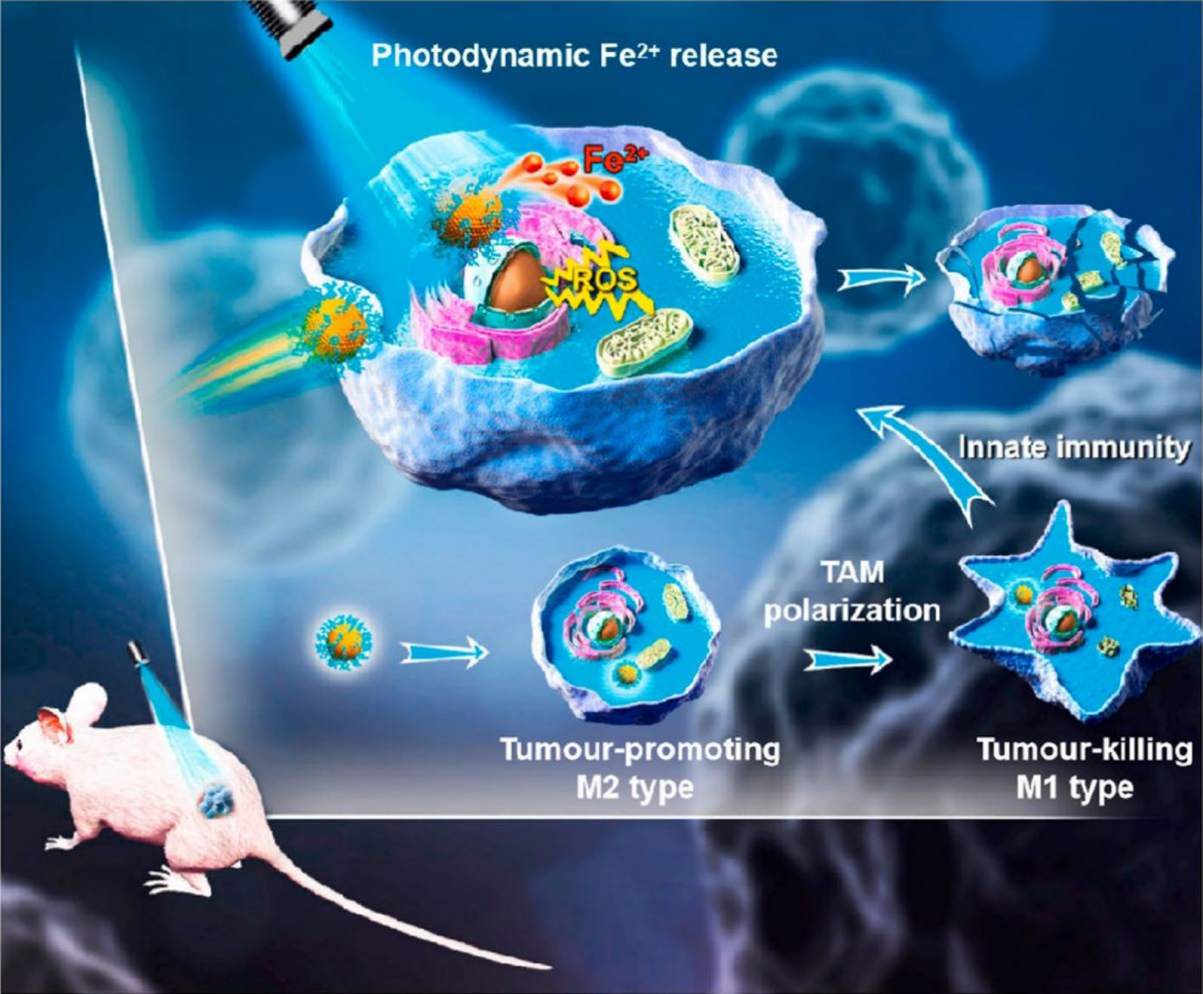
Reproduced with permission from [111] @Copyright 2022, Elsevier Ltd

Biocompatible PEG-coated ferrihydrite nanoparticles (PEG-Fns) show the therapeutic potential of ferrihydrite to combine ROS-based CDT, PDT, and M1-activating immunotherapy with Fe^2+^ as the connecting point (Fig. 5) [110]. Triggered by visible blue light instead of UV light, the photoresponsive system generates Fe^2+^ and ROS and inhibits GPX4, which leads to apoptosis- and ferroptosis-dependent cancer cell proliferation inhibition [111]. TAM polarization from the tumor-promoting M2 type to the tumor-killing M1 type is simultaneously activated by PEG-Fns, which concomitantly inhibits tumor growth and prevents pulmonary metastasis in vivo [112].

**Fig. 6:**
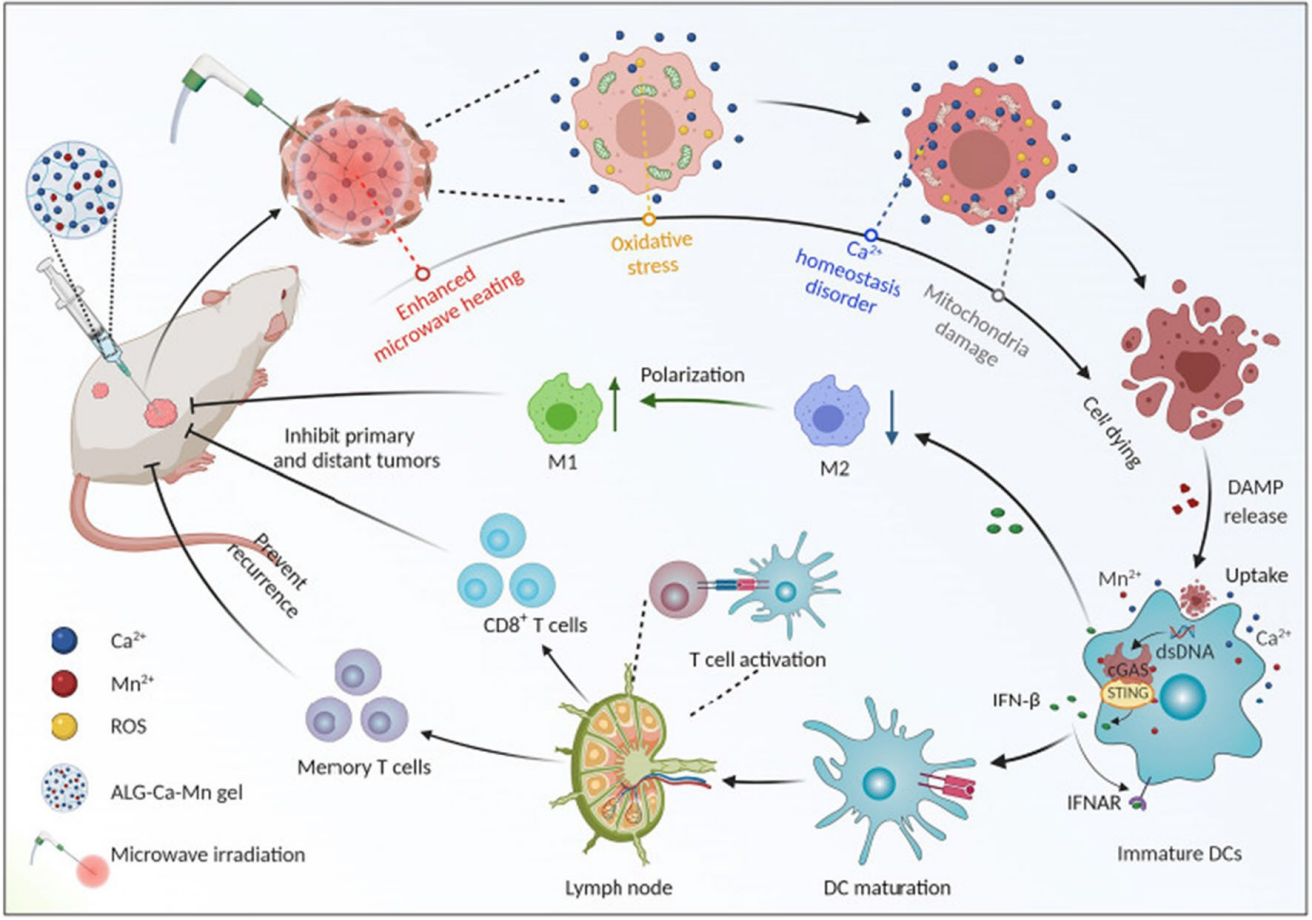
Reproduced with permission from [138] @Copyright 2022, American Association for the Advancement of Science

### Gold nanoparticle (AuNP)-based nanoimmune activators

AuNPs are applied in light-triggered thermal therapy in the form of gold–gold sulfide nanoparticles, hollow gold nanoshells, and gold–silica nanoshells because of their high absorption property in the second near-infrared (NIR) light window, which can lead to hyperthermia in tumor sites for PTT/PDT and facilitate drug release [113, 114].

Intravenous injection of CpG immunostimulants by AuNP delivery systems can lead to better accumulation of nanoparticles in the reticuloendothelial system (RES) [115]. Moreover, AuNPs can induce macrophage and DC infiltration, which can inhibit cancer development in the context of B16-ovalbumin (OVA) [116, 117]. This provides novel insight and an ideal platform for cancer vaccine design; in this context, phagocytosis by macrophages and DCs is favorable for antigen transportation to increase the overall amount of the vaccine delivered to APCs [117–119].

Mangiferin-functionalized gold nanoparticulate agents (MGF-AuNPs) were designed to treat prostate cancer by elevating the levels of certain antitumor cytokines, such as IL-12 and TNF-α, and simultaneously reducing the levels of protumor cytokines, such as IL-10 and IL-6, which target macrophages in the spleen via NF-kB signaling pathway activation [120]. Immunomodulatory metal nanoparticles transform protumor M2 macrophages into antitumor M1 macrophages, substantially improving patients’ therapeutic outcomes [121].

An innovative immunological AuNP (AuNP@B16F10) has been developed; melanoma B16F10 cells were first employed to generate AuNPs, and then these cells shed nanoparticles with retained tumor antigen-trapped vesicles into the extracellular environment. AuNP@DC_B16F10_ particles were further constructed by introducing the nanoparticles into DCs, which can induce hyperthermia and provoke antitumor immune responses for combinatorial PTT and immunotherapy [122].

### Ag_2_S-based and sliver nanoparticle (AgNP) nanoimmune activators

Ag_2_S with an appropriate size can elicit a photothermal-based tumor-killing effect. At the same time, Ag_2_S is applied in imaging due to its ideal tissue penetration ability to provide more detailed cancer diagnosis information [123, 124]. An NIR light-responsive NO delivery system was developed for the controlled and precise release of NO to hypoxic tumors during radiotherapy [125].

Ag_2_S quantum dots (QDs) coupled with an efficient NO source, tert-butyl nitrite, are able to generate NO under NIR irradiation induced by the thermal effect [126]. These Ag_2_S@BSA-SNO NPs can ameliorate the immunosuppressive TME by significantly enhancing anti-PD-L1 immune checkpoint blockade therapy. Multifunctional cancer radioimmunotherapy based on Ag_2_S NO delivery platforms showed a 100% survival rate, which remarkably maximized radiotherapy effects to inhibit tumor growth in a CT26 tumor model [127].

Among the gut microbiota, Fusobacterium nucleatum (Fn) selectively increases the proportion of MDSCs in CRC. M13@Ag was designed by assembling AgNPs electrostatically on the surface capsid protein M13 to specifically clear Fn and remodel the suppressive TME by activating APCs to further strengthen the host immune system. Combined with immune checkpoint inhibitors (α-PD1) or chemotherapeutics (FOLFIRI), M13@Ag prolonged overall mouse survival in the orthotopic CRC model to a greater extent [128].

### Copper and CuS-based nanoimmune activators

Intratumoral copper levels influence PD-L1 expression in cancer cells and contribute to cancer immune evasion. The expression levels of the major copper influx transporter copper transporter 1 (CTR-1) and PD-L1 are closely related across many cancers but not in corresponding normal tissues [101].

The treatment prognosis, sensitivity to chemotherapy based on immunophenotype, and immunotherapy response can be predicted based on cuproptosis-related genes in bladder cancer as the infiltrating landscape of immune cells (especially T cells and DCs) induce a nonapoptotic type of programmed cell death caused by excess copper [129]. Further application remains to be reported in triple-negative breast cancer (TNBC) [130].

Featuring the high photothermal conversion efficiency of copper sulfide (CuS) nanoparticles, abundant deposition inside the large pores of dendritic large-pore mesoporous silica nanoparticles (DLMSNs), and the immune adjuvant resiquimod (R848), AM@DLMSN@CuS/R848 has been incorporated to treat TNBC by combining photothermal ablation and immune remodeling through tumor vaccination and T lymphocyte activation [131].

In addition, cancer cell-macrophage hybrid membrane-coated, NIR-responsive, hollow CuS nanoparticles can encapsulate sorafenib and be surface-modified with anti-VEGFR (CuS-SF@CMV NPs); the other ataxia telangiectasia mutated (ATM) inhibitor-loaded hollow-structured CuS NPs with surface modification with anti-TGF-β have the function of target specificity and immune activation (CuS-ATMi@TGF-βNPs); both these structures show application potential in hepatocellular carcinoma (HCC) as they synergize with PTT, chemotherapy and immunotherapy [132].

### Zinc and ZnS-based nanoimmune activators

Zn^2+^ is essential for innate and adaptive immune activation and proper function of innate and adaptive immune cells, including the cytotoxic activity of NK cells and T cells [133]. Taking advantage of the excellent bioavailability of encapsulated ionotropic drugs, hesperidin-loaded Zn^2+^@ sodium alginate/pectin (SA/PCT) nanocomposites were designed to inhibit the proliferation of colon carcinoma cells and induce apoptosis under in vitro conditions [134].

In addition, the innovative delivery system FEGCG/Zn is integrated with fluorinated-coordinative-epigallocatechin gallate (EGCG) and Zn^2+^. The robust therapeutic effects of FEGCG/Zn depend on excellent delivery of small interfering RNA of PD-L1 (siPD-L1) and further siPD-L1 accumulation in tumors, which enhances antitumor immunotherapy through alleviation of T-cell exhaustion by regulating PD-L1 expression in tumor cells [135].

Another new ZnNP is ZnPP@MSN-RGDyK: zinc protoporphyrin (ZnPP)-loaded mesoporous silicon nanoparticles are combined with a new PD-L1 inhibitor RGDyK, which was reported recently with high photodynamic therapy efficiency, excellent immunotherapeutic effects and precise integrin β3 (β3-int) targeting in an NSCLC-SM mouse model [136].

ZnS@BSA (bovine serum albumin) nanoclusters have been synthesized to activate cGAS-STING signals in mice, promoting the infiltration of CD8^+^ T cells at the tumor site and cross-presentation of DCs, which can improve immunotherapy efficacy against HCC [137]. Systemic evaluation of in vitro cytotoxicity demonstrated the good biocompatibility of the proposed BSA-conjugated ZnS nanoparticles (Table 2). These studies suggest that the prepared BSA-conjugated ZnS nanoparticles are promising for future applications in antitumor immunity and biomedical engineering.

**Table 2:** Several metallic nanoparticles have been applied in cancer therapy for immune modulation

Nanoparticle	Metallic material	Delivery technology	Additional agents	Cancer type	Immune modulation	Effectiveness	Refs.
PL/APMP-DOX	Mn_3_ (PO_4_)_2_(APMP)	Lipid bilayer coating (PL)	Doxorubicin (DOX)Generate DNA damage	Breast cancer (4T1 cells)	Augments cGAS/STING activity	Tumor targeting; DC maturation; T cell infiltration; NK recruitment; Type I-IFNs,TNF-a↑	[80]
H-MnO_2_-PEG/C&D	Hollow MnO_2_	Polyethylene-glycol (PEG)	Photodynamic agent chlorine e6 (Ce6); DOX	Breast cancer (4T1 cells)	Enhances macrophages infiltration; facilitates M2/M1 polarization	T1WI MRI imaging; H_2_O_2_ decomposition; metastasis inhibition; combined chemo-PDT	[81]
Mn@CaCO_3_/ICG@siRNA	Walnut-like MnO_2_ NPs	PH-response cover layer of CaCO_3_	PD-L1-targeting siRNA; ICG	Lung cancer (Lewis cells)	Improves CTL-mediated antitumor immunities	Enhances PDT effect; relieves tumor hypoxia; silences PD-L1 gene; DC infiltration, effector↑	[82]
CpG@PLGA-PLLmPEG/SPIO (MINPs)	Superparamagnetic iron oxide (SPIO)	mPEG-PLGA-PLL triblock copolymer	Immunoadjuvant CpG-ODNs Activate TLR9	Breast cancer (4T1 cells)	Activates DCs; induces TNF-α, IL-6 and IFN-γ secretion	Magnetic targeting; synergistic PTT; precise MR/PA image; photo-immunotherapy	[85]
PEG-coated ferrihydrite nanoparticles (PEG-Fns)	Fe(NO_3_)_3_·9H_2_O	Biocompatible PEG-coating	/	Lung cancer (SCC-7 cells)	Induces M1 macrophage polarization	Promotes ROS-based CDT; increased oxidative stress; apoptosis/ferroptosis↑ metastasis prevention	[87]
Fe^2+^/siRNA/PDA nanoparticles (FesiRNAPNPs)	FeCl_2_	Polydopamine (PDA)	Fe^2+^ chelator -GMP; GAPDH siRNA	Colon cancer (CT26 cells)	ROS generation & GSH consumption in the TME leads to tumor killing	Accumulation of LPO; Fe^2+^-induced ferroptosis Transformation of H_2_O_2_ into ·OH Suppression of tumor growth	[88]
PolyA-CpG-AuNPs	Nano-gold (AuNPs)	Poly-adenine (polyA) tail	Immunoadjuvant CpG-ODNs	Leukemia (RAW264.7)	Immune- stimulatory activity via TLR9	Efficient delivery; induces expression of proinflammatory cytokines	[91]
AuNP4	HAuCl_4_ · 3H_2_O	Monolayer Mercaptosuc-cinic acid (MSA)	Radiation	Breast cancer (MCF7)	Tumor neoantigen presentation, cytokine secretion; priming of host antitumor T cells	Enhances RT induced ICD tumor growth delay; TME immunogenicity↑ Radiosensitization	[92]
Mangiferin functionalized (MGF)-AuNPs	NaAuCl_4_	Mangiferin (polyphenol:Dglucoside)	/	prostate cancer (PC-3 cells)	Antitumor cytokines (IL-12 and TNF-α)↑; IL-10, IL-6↓	Targets macrophages via the NF-kB pathway; anti-angiogenesis agent; reprograms M2 macrophages to M1 macrophages	[96]
AuNP@B16F10	HAuCl_4_	Exocytosis of B16F10/ DC 2.4	Coincubation with DC2.4; exposure to UV irradiation; NIR	Melanoma (B16F10 cells)	Provides a pro-inflammatory immune environment	Promoted DC maturation Tumor-infiltrating lymphocytes↑; induces hyperthermia; anti-metastasis effect	[98]
Silver/silver sulfide Janus nanoparticle (Ag/Ag_2_S JNP)	AgNO_3_	PEGylated	H_2_O_2;_ NIR-II	Breast cancer (MCF7)	PTT; noninvasive location and diagnosis in vivo	H_2_O_2_-activated imaging; NIR-II fluorescence switching specifically activated by H_2_O_2_	[101]
AM@DLMSN@ CuS/R848	Copper sulfide (CuS)	Dendritic large-pore mesoporous silica nanoparticles (DLMSNs)	Anti-PD-1 peptide AUNP-12; Resiquimod (R848)(TLR7/8 agonist); NIR	Breast cancer (4T1 cells)	Photothermal ablation induced ICD	High photothermal conversion efficiency; PTT agent suppressing TNBC growth and metastasis; block the PD-1/PD-L1 pathway	[108]
CuS-sorafenib-anti-VEGFR antibodies (CuS-SF@CMV)	Hollow-CuS	Cancer cell-macrophage hybrid membrane-coating	Sorafenib; anti-VEGFR antibodies	Hepatocell-ular carcinoma (HepG2 cells)	Localizes to the homotypic cells and escapes immune cell-mediated elimination	Inhibits tumor cell proliferation and angiogenesis via the Ras/Raf/MEK/ERK and PI3K/AKT pathways	[109]
ZnS@BSA	ZnS	BSA (bovine serum albumin)	Generated H_2_S	Hepatoma	Activates cGAS/STING pathway	ROS production; infiltration of CD8^+^ T cells, cross-presentation of DCs↑	[114]
Zn^2+^@ SA/PCT	ZnCl_2_	Hesperidin	Sodium alginate (SA), pectin(PCT)	Colon cancer (HCT116)	Induces apoptosis via excessive generation of ROS	Inhibited proliferation of colon carcinoma cell and induced apoptosis in in vitro condition	[111]

### Synergistic application of multiple metal ions

Sodium alginate (ALG)-Ca-Mn gel is an innovative microwave ablation (MWA) nanomaterial developed for local thermal ablation of tumors using the heat generated by the intense oscillation of metal ions under microwave exposure. The combination of increased [Ca^2+^]e (e.g., 4 mM) with mild hyperthermia (43 °C) induces the immunogenic cell death (ICD) of cancer cells by eliciting intracellular Ca^2+^ overloading as well as mitochondrial dysfunction, which leads to effective cancer cell killing. Moreover, Mn^2+^ can elicit potent innate and adaptive antitumor immunity via cGAS-STING activation (Fig. 6). This immune nanoactivator based on metal ions shows great capacity to improve MWA treatment effectiveness [138]. Recently, mPEG-b-PHEP incorporating IR780 dye and manganese zinc sulfide nanoparticles (ZMS) (PP_IR780-ZMS_) was established. This thermally responsive biopolymer micelle with Zn_0.43_Mn_0.57S2_ nanoparticles can readily induce antitumor immunity. With NIR induced by IR780 dye, the precise release of ZMS nanoparticles produces DAMPs and then boosts ICD. In particular, Mn^2+^ can not only generate ROS but can also enhance immune filtration in neoplastic foci, which reverses the suppressive phenotype of the TME to allow effects against the primary tumor and pulmonary metastases with safe systemic cytokine expression via the synergism of PDT/CDT and immunotherapy [139].

GNRs@SiO_2_@MnO_2_@MDSCs (GSMM) is obtained by combining gold nanorods (GNRs) with MnO_2_, which further disguises the MDSC membrane on its surface. Mn^2+^ catalyzes H_2_O_2_ into ·OH for CDT, leading to the activation of cGAS-STING but also directly acts on STING, inducing the secretion of type-I interferons, proinflammatory cytokines and chemokines. Additionally, PTT and CDT-mediated ICD of tumor cells can further enhance antitumor immunity via exposure to calreticulin (CRT), high mobility group protein B1 (HMGB1) and adenosine triphosphate (ATP) [140].

## Discussion

As we have generally described, the metabolism of several metal ions in the TME, especially concerning antitumor immunity, has been well established. The slightest change in their extra/intracellular concentration results in amplified effects by signaling cascades that determine cell fate and immunity status [141, 142]. Moreover, the self-regulation of metal ion metabolism and application of nanometallic materials influence the network of antitumor immunity through various pathways. Because metal ions influence the fate of cancer cells and participate in both innate and adaptive immunity, they are widely applied in antitumor treatments [143]. However, there are some issues that remain to be resolved.

First, the delivery system and administration route should be taken into account. For example, some administration routes, including intranasal, intravenous, and intratumoral routes, induce systemic antitumor responses, which might be applicable to cases of widely metastatic cancers, whereas local injection might be more suitable for early-stage cancers or certain types, such as retinoblastoma [144].

Secondly, unwanted damage to normal tissues should be avoided by designing selective metal ion-based immune activators in cancer cells. In addition, in consideration of tumor diversity and heterogeneity, ultrasensitive nanomaterials should be designed to distinguish the tumor margin from normal tissues based on enzymatic activity and acidity [145].

More importantly, specific drug delivery vehicles are essential to realize precise delivery of biomimetic drugs in diverse tumor microenvironments with various biological barriers. The design of a loading platform depends on the application of nanotechnology in the domain of physiopathology concerned with metal and tumor physio-biochemical characteristics.

Despite the fact that metal ion application is considered to be a promising cancer therapy without the introduction of exogenous substances, we should focus on evaluation before treatment and monitoring after treatment because metal ions can directly affect cell excitability and manifest cytotoxicity beyond a certain dosage [139]. As potential agonists of the immune system, we should discover a balance between immune surveillance and homeostasis to avoid hyperinflammatory reactions that will be a double-edged sword for cancer therapy.
